# *Puumala hantavirus* Infection in Humans and in the Reservoir Host, Ardennes Region, France

**DOI:** 10.3201/eid0812.010518

**Published:** 2002-12

**Authors:** F. Sauvage, C. Penalba, P. Vuillaume, F. Boue, D. Coudrier, D. Pontier, M. Artois

**Affiliations:** * Université C. Bernard Lyon 1, France; † Centre Hospitalier, Charleville-Mézières, France; ‡ Entente Interdépartementale de Lutte contre la Rage, Malzéville, France; § AFSSA Nancy, Domaine de Pixerécourt, Malzéville, France; # Institut Pasteur, Paris, France; ** Ecole nationale vétérinaire de Lyon, Marcy l'Etoile, France

**Keywords:** Bank vole, indirect transmission, Hantavirus, Nephropathia epidemica, Puumala, HFRS

## Abstract

We compared the occurrence of nephropathia epidemica cases, over a multi-annual population cycle, in northeastern France with the hantavirus serology for bank voles captured in the same area. We discuss hypotheses to explain the pattern of infection in both humans and rodents and their synchrony.

In Eurasia, hantaviruses (family *Bunyaviridae*) are the etiologic agents of hemorrhagic fever with renal syndrome (HFRS) in humans (1). In France, the HFRS-endemic area is the northeastern quarter of the country (2–4). The Ardennes massif at the Belgian border hosted 244 recorded human cases during 1991–1999 and accounted for two thirds of the total number of French cases during the 1996–1999 regional bank vole (*Clethrionomys glareolus*) demographic cycle. Historically, 40% of all recorded French cases occur in this region. Human cases of nephropathia epidemica (NE), a milder form of HFRS caused by *Puumala virus* (PUUV), are routinely recorded at the Centre Hospitalier Régional (CHR) of Charleville, (Ardennes) France, which has the largest number of clinical cases in the country. Epidemic outbreaks of acute infection have been observed every third year since 1991. We hypothesize that the risk of human infection results from an increase in the mass shedding of virus, after a population increase of infected voles over a threshold density. In this case, the prevalence of infection in rodents may rise some time before the outbreak occurs in humans.

To test this hypothesis, we set up a surveillance protocol to investigate if increased densities of bank vole populations would amplify anti-PUUV antibody prevalence in the reservoir, reflecting an increased risk for infection by human beings. We determined the prevalence of anti-hantavirus antibodies in a population of bank voles, the rodent reservoir (5), within the disease-endemic area. During the 3 years before the last peak of disease (1997–1999), we monitored bank vole populations by trapping and screening antibodies. Animals were trapped in the Elan and Hazelles forests near the city of Charleville by using 100-m trap lines, each containing 34 nonbaited small rodent INRA box traps (6) in the spring, summer, and autumn of 1997, 1998 (plus one session in December 1998), and 1999. The forests were divided into equal sectors, and 12 trap lines by forest (13 in 1997) were set in randomly chosen sectors for each capture session. Traps were checked daily for 3 successive days, and captured rodents were removed for further virologic studies. If lines were reset in the same sector on successive occasions, they were moved to reduce bias caused by the removal of animals trapped in the previous session.

Serum samples of blood from the trapped animals were withdrawn by cardiac puncture and evaluated by enzyme-linked immunosorbent assay (ELISA) on plaques directly coated with antigen from cells infected with PUUV or controls, lysed in triton-borate buffer, and then sonicated. Antibody uptake was estimated with peroxidase-tagged anti-mouse immunoglobulin (Ig) G, which cross-reacts with bank vole Ig (not shown). Positive serum samples were confirmed by immunofluorescence on PUUV or *Haantan* virus-infected Vero E6 cells at serial doubling dilutions. We did not use direct detection of the virus by reverse transcriptase polymerase chain reaction (RT-PCR). Therefore, a discrepancy between our estimates of infected voles and the true number of potentially infectious animals may have occurred but should not affect the temporal trends over a 3-month intervals (7,8).

During the study, we observed a fourfold increase in the density index of the monitored vole population, with seasonal fluctuations (highest in September and lowest in spring). A total of 550 animals were trapped during 25,092 trap nights for an overall trap success rate of 2.19% (3.25% in Elan, with 408 captures and 1.13% in Hazelles, with 142 captures). Five species of rodents and two species of insectivores were captured. Overall, *C. glareolus* was most commonly collected (49.8%); however, the proportion of the different species varied greatly between the two forests: bank voles accounted for 57.6% of captures in Elan but only for 27.5% of captures in Hazelles (χ²=13.65, p = 2.10^-4^), where they were overtaken in frequency by *Apodemus flavicollis* (29.6%). Twenty-nine rodents were seropositive for hantaviruses; 25 were bank voles (23 in Elan and 2 in Hazelles) and 4 were *A.* *sylvaticus* (3 in Elan and 1 in Hazelles). Seropositive wood mice were detected during the peak of prevalence in bank voles, which suggests a spillover infection similar to that seen in humans. We focused on bank voles in Elan because the number of infected voles from Hazelles is too small to allow statistical analysis. Among the 235 bank voles captured, 113 were male, 119 were female, and 3 were undetermined. The overall seroprevalence was 9.8% (12 seropositive voles), with 13 males (prevalence of 11.5%), 9 females (7.6%), and 1 undetermined. Prevalence did not differ between sexes (χ²=0.64, p=0.42). Hantavirus antibody prevalence reached a maximum of 29% (4 of 14 bank voles tested) during the spring of 1999 from 8.9% (5/56) in the previous fall. Prevalence then fell to 11% (4/36) the next summer and remained at this level (7/76) until the population peak in September 1999. One might hypothesize that the amount of virus available for human contamination reached its highest level between September 1998 and September 1999.

We observed an irregular distribution of seropositive animals among the captures from the Elan forest (Figure 1); many trap lines did not have positive voles from a large number of captures, whereas others had higher rates (up to 3 of 3 captured voles). No clear correlation exists between host density (as estimated by capture frequency) and seropositivity, but seropositive animals were more often taken on northerly facing trap lines (χ²=12.68, p=4.10^-4^) than southerly facing ones (χ²=0.86, p=0.35). This difference is probably due to the higher humidity of northerly facing slopes, which are less often exposed to the sun. Verhagen et al. (9) have reported that the probability that a bank vole will be infected increases with the humidity of its territory. The lower number of observations from the Hazelles forest also showed a variation in population density and antibody prevalence in synchrony with the Elan voles.

**Figure 1:**
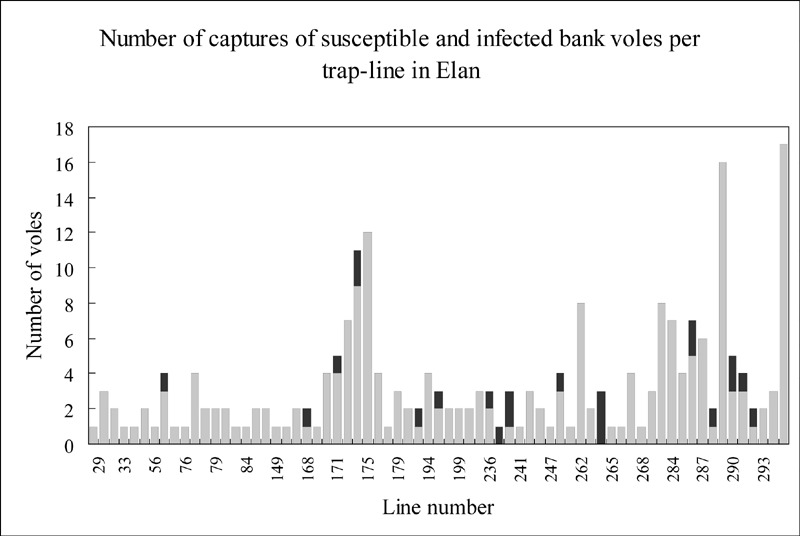
Number of captured bank voles (susceptible in light grey, seropositive in black) per trap line in Elan.

At least 40 human cases of NE (range 40–74) were recorded at the Charleville CHR in 1993, 1996, and 1999, whereas no more than 14 NE cases were seen in the intervening years (Figure 2). If the sampled rodents are representative of the whole reservoir population to which humans are exposed, our findings suggest a synchrony of infection rates in humans and reservoir rodents over the 3-year epidemiologic cycle. In accordance with previous records, the greatest number of HFRS cases were registered at Charleville CHR during the periods of highest prevalence in the reservoir host (1993, 1996, and 1999). Provided that the data from this preliminary study are accurate, the temporal correlation between infection rates in human victims and in the reservoir host (Spearman's rank correlation, z=2.55, p=0.01 p= 0.86) strongly suggests a common process of infection. The assumption that the vole demographic population peak precedes the epidemic outbreak in humans is not supported by our data. If our study is representative of the actual situation, our results suggest that the maximum infection rate is reached simultaneously in both the human and reservoir hosts. These results could be explained if the proportion of newly infected voles is more important than the total number of infected voles. In fact, the amount of virus shed during the first month of infection is far higher than during the consecutive chronic phase (10). In this case, the increased mass shedding of virus would not necessitate a very high prevalence. To attain the observed high proportion of infected voles for less than 1 month, even with a stable prevalence, the transmission between voles must be rapid in the increasing population.

**Figure 2:**
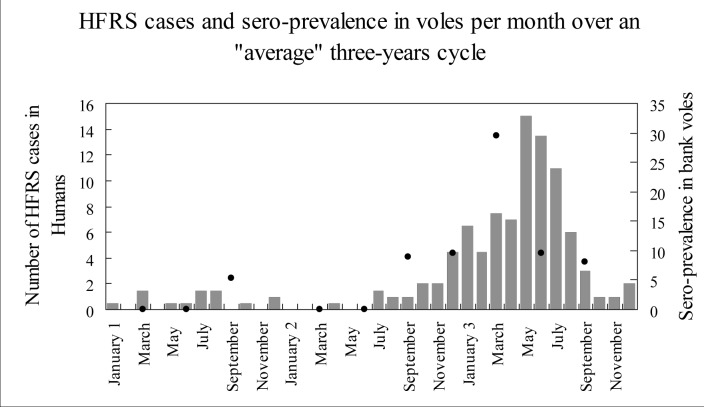
Temporal correspondence of reservoir contamination and of human cases of nephropathia epidemica over a typical 3-year cycle. Grey bars: the number of HFRS cases in humans per month for the Ardennes region (France) from 1991 to 1996; black points: observed hantavirus antibody prevalence in bank voles by trapping session in Elan forest over the same period. Right scale: percentage of seropositive voles in the trapped sample.

We hypothesize that the mechanism of virus circulation is related to the social structure of the reservoir: bank voles are territorial and avoid encounters with conspecifics during the breeding season but share nests during winter. Direct transmission seems difficult during the reproductive season. We have previously examined a possible role of the environment in the survival of the virus outside its host to explain its observed patchy distribution (11). Human contamination occurs mainly by this indirect route (12). The bank vole social component, in combination with an indirect transmission route, can explain the rapid spread of infection through a population of increased density. The voles may become infected through the sniffing of contaminated excreta marks, even though these animals avoid direct encounters. This activity could result in a high proportion of newly contaminated voles. A change in the population dynamics of the bank vole reservoir, leading to a low incidence of newly infected individuals as seen in the more stable populations, could explain the transition from an epidemic to a sporadic pattern of HFRS in regions of France south of the Ardennes (2). This hypothesis will be tested in ongoing epidemiologic studies.
